# The impact of perceived teacher support on Chinese junior high school students’ academic self-efficacy: The mediating roles of achievement goals and academic emotions

**DOI:** 10.3389/fpsyg.2022.1028722

**Published:** 2022-11-18

**Authors:** Xiaodan Ren, Bin Jing, Hongxia Li, Changcheng Wu

**Affiliations:** School of Computer Science, Sichuan Normal University, Chengdu, China

**Keywords:** perceived teacher support, academic self-efficacy, achievement goal, academic emotion, mediating role

## Abstract

**Introduction:**

Teacher support is an important external factor that influences students academic self-efficacy, however, the mechanisms of the two factors are not yet fully explored. The purpose of this study was to investigate whether achievement goals and academic emotions could play a chain mediating role between perceived teacher support and academic self-efficacy.

**Methods:**

The study sample was made up of 1,074 Chinese junior high school students, and three structural equation models were constructed using data collected from on questionnaires.

**Results:**

The findings suggest that achievement goals and academic emotions can mediate the relationship between perceived teacher support and academic selfefficacy. Further analysis revealed that achievement goals and academic emotions may play a chain mediating role between perceived teacher support and academic selfefficacy.

**Discussion:**

These findings provide reference points for further refinement of the mechanism of the role of perceived teacher support on academic self-efficacy. They also serve to remind the teacher on the front line to focus on how to provide adequate teacher support to students in the context of online education, especially with regard to students academic emotions.

## Introduction

According to social cognitive theory (SCT), self-efficacy is generated in four ways: enactive mastery experiences, vicarious (observational) experiences, social persuasion, and physiological, psychological states (Bandura, 1999; van Dinther et al., 2011). Enactive mastery experiences are the most powerful source of creating a sense of self-efficacy, but conclusions that support this may be somewhat biased (van Dinther et al., 2011). Junior high school students are in a semi-naive and semi-mature stage and do not think thoroughly enough, resulting in them needing the help and support of significant others (e.g., teachers or parents; Qiao et al., 2013). Research has shown that various forms of feedback from teachers can improve students’ academic performance and autonomous learning skills, which may enhance their self-efficacy (Zhang et al., 2022). In the Chinese educational context, junior high school students have reached the first major crossroads of their lives, facing pressures of further education while experiencing fluctuating mental and emotional states (Qiao et al., 2013). In general, positive emotions enhance individuals’ sense of self-efficacy while negative emotions weaken it, and people tend to rely on these emotional states to assess their abilities by perceiving and interpreting this information (Pajares, 1997). Defined as the Pygmalion effect, when students receive both external recognition and high expectations, they tend to change their self-positioning and goals, and are more likely to succeed in the future (Szumski and Karwowski, 2019). In a learning environment, teacher support seems a likely way to predict changes in individual students’ self-efficacy due to teachers’ opportunity to influence students’ achievement goal orientation.

Several studies have already explored the mechanisms of action between perceived teacher support and self-efficacy, research on the mechanisms underlying the role of academic goals and academic emotions with them is not clear enough. Therefore, the purpose of this study was to investigate whether achievement goals and academic emotions play a chain intermediary role between perceived teacher support and self-efficacy. The findings can enrich relevant theoretical research and provide practical references for how teachers can support the academic and psychological development of junior high school students.

## Literature review

### Perceived teacher support and academic self-efficacy

Academic self-efficacy refers to the confidence that individuals believe they can successfully complete learning tasks based on their judgment of their own abilities, attitudes and past experiences (Schunk, 1991; Lorsbach and Jinks, 1999). And academic self-efficacy can be broken down into learning competence and learning behavior. Students’ academic self-efficacy may be influenced by the comparative social assessment used by teachers and the comparison of one’s personal knowledge with that of other students (Olani et al., 2010). According to feedback channel 2 in self-determination theory, self-efficacy can also have a converse feedback role (Deci and Ryan, 1980). For example, individuals with a high level of academic self-efficacy tend to perform better in difficult tasks and activities and vice versa (Mercer et al., 2011).

According to self-determination theory (SDT; Deci and Ryan, 1980), individual development depends on three basic psychological needs: competence, autonomy, and relatedness. Teacher support is an important factor in learning environment for students in the SDT model, such external information input can influence students’ goal selection by triggering students’ motivation subsystem, especially for junior high school students who are still unable to make rational choices. For example, when students receive adequate teacher support, they are motivated to learn and thus produce positive outcomes (Jang et al., 2009). Perceived teacher support is defined as students’ perceptions that their teachers are concerned about them and will assist them if needed through academic support, emotional support, and competence support (Trickett and Moos, 1973; Liu et al., 2021). Research has shown that students who feel more teacher support in math classes had more positive attitudes and greater self-efficacy for learning math (Rice et al., 2013). However, when the learning environment lacks adequate support, students are less likely to translate their academic interests into goals and actions, leading to a reduction in students’ academic self-efficacy (Olani et al., 2010). Numerous studies have shown that perceived teacher support can positively predict academic self-efficacy (Liu et al., 2021). Support as an external factor requires the development of internal factors in order to play a facilitating role (Helgeson and Lopez, 2010).

### Mediating role of achievement goals and academic emotions

Perceived teacher support can positively predict autonomy motivation, and autonomy motivation can positively predict relaxation, which then positively predicts creative self-efficacy (Liu et al., 2021). Self-efficacy and positive emotions play roles in the relationships between teacher support and math engagement (Liu et al., 2017). Furthermore, teacher support has been shown to positively predict academic self-efficacy, and academic self-efficacy can then positively predict positive emotions (i.e., enjoyment and relief), as well as positively predicting sub-dimensions of engagement (i.e., cognitive engagement, behavior engagement, and emotional engagement; Liu et al., 2017). To our knowledge, no studies have yet investigated whether achievement goals and academic emotions can play a chain mediating role between perceived teacher support and self-efficacy. And currently, scholars have yet to form a systematic understanding of the relationships between common variables such as perceived teacher support, self-efficacy, engagement, academic emotion, and achievement goals. Therefore, to build a systematic and comprehensive functional system, these relationships must be explored further. This study aimed to provide empirical support for the investigation of these relationships and to provide some guidance for teaching practice.

#### Achievement goals as the mediators

To our knowledge, there is no uniform definition of achievement goals. Therefore, this study has chosen to use Pintrich’s (2000) definition of achievement goals: “cognitive representations of the reasons and purposes for which individuals pursue achievement goals.” The current research adopted the 2 × 2 achievement goal framework to divide achievement goals into performance-approach, performance-avoidance, mastery-approach, and mastery-avoidance goals (Liu et al., 2020a). Different goal orientations will lead to different affective, cognitive, and behavioral results (Daumiller and Dresel, 2020). For example, adopting mastery-oriented goals is related to various adaptive outcomes (e.g., positive emotions, high self-efficacy, low burnout, effective self-regulation, and learning engagement; Liu et al., 2020a). Conversely, adopting performance-oriented goals is associated with maladaptive outcomes (e.g., anxiety, low self-efficacy, poor academic achievement, surface learning, and disengagement; Liu et al., 2020a).

Junior high school students are at an important stage in the formation of their outlook on life and are both semi-naive and semi-mature, needing more support from teachers, parents, and friends (Qiao et al., 2013). Teachers, as significant others for students, can shape students’ academic values and behaviors (Régner et al., 2009). Empirical studies have shown that perceived teacher support had an impact on students’ achievement goal orientation (Vansteenkiste et al., 2004). Specifically, students are more likely to favor mastery-oriented goals when they perceive academic support from teachers (Régner et al., 2009). Because individuals who adopt mastery-oriented goals focus on absolute or intrinsic criteria and believe that effort will lead to success, they tend to have a high sense of self-efficacy (Huang, 2016). According to SDT, teachers’ interaction style may support or hinder students’ basic psychological needs, thus affecting students’ motivation and development (Vermote et al., 2020). In addition, emotional support from teachers (e.g., enthusiasm) will affect the motivation of students with mastery orientation and low avoidance goals in mathematics learning (Keller et al., 2016). Conversely, self-efficacy is compromised when one tries one’s best without achieving tangible results (Huang, 2016). Teachers play many roles for their students, one of which being a supervisor. Students are more likely to favor performance-oriented goals when they perceive supervisory attributes from their teachers (Régner et al., 2009). Motivation that arises from approach-oriented goals usually leads to positive learning experiences, and these positive outcomes are thought to be associated with a higher sense of self-efficacy (Huang, 2016). In contrast, avoidance-oriented goals produce behaviors that point to outcomes that are difficult to achieve, and individuals are prone to stressful learning experiences (Elliot, 2006). Thus, avoidance-oriented goals are thought to be associated with lower self-efficacy (Huang, 2016).

To our knowledge, studies have shown that competence needs directly or indirectly predict mastery of achievement goals through self-efficacy (Diseth et al., 2012). However, little current study has explored whether achievement goals can mediate the relationship between perceived teacher support and self-efficacy. In the current study, we speculated that achievement goals might play an intermediary role between perceived teacher support and self-efficacy. Thus, the current study was intended to further investigate whether achievement goals play a mediating role between perceived teacher support and self-efficacy.

#### Academic emotions as the mediators

Academic emotions refer to the various emotions of achievement that students experience in the school environment, particularly those associated with success or failure (Pekrun et al., 2002). Academic emotions can be divided into two dimensions: positive and negative (Pekrun et al., 2011). According to the broaden-and-build theory, positive emotions expand the range of activity of the individual’s transient mind, which is essential for creativity (Fredrickson, 2001). This can then help individuals build enduring internal resources, from physical and intellectual resources to social and psychological outcomes (Fredrickson, 2001). Meanwhile, negative emotions generally lead to a sense of crisis and urgency which can limit cognitive activity (Zhen et al., 2017). However, there is a positive side to negative emotions, specifically that while negative emotions such as boredom may demotivate most students, they can also motivate individuals to leave their current environment or change their behaviors (Artino and Durning, 2011; Liu et al., 2021). Even anxiety can ultimately facilitate learning by motivating students’ actions, especially in those with a high sense of self-efficacy (Artino and Durning, 2011).

According to the control-value theory (CVT; Pekrun, 2006), the environmental characteristics of transmitting information related to controllability and academic value are crucial to students’ emotions, and autonomous support is an important variable. Support from teachers can affect students’ sense of control and value measurement of learning tasks, thus affecting the change of students’ academic emotions. According to the feedback path between emotion and evaluation, emotion will in turn affect the sense of control value of the current task. A meta-analysis showed that teacher support was significantly positively associated with positive academic emotions and negatively associated with negative academic emotions (Lei et al., 2017). Junior high school students are emotionally sensitive and changeable, and expect to get attention from their elders, including teachers and parents (Qiao et al., 2013). In response to teachers’ negative responses students can develop negative emotions such as boredom and negativity (Assor et al., 2005). Such negative emotions can negatively predict learning satisfaction and may damage students’ sense of self-efficacy (Wu et al., 2021). Conversely, when students receive timely and honest feedback from teachers, they are more engaged in their learning and thus achieve better results (Zhao, 2014). SCT suggests that emotional arousal affects individuals’ self-efficacy judgments (Bandura et al., 1997). The induction of positive emotions has been shown to increase university students’ level of academic self-efficacy, while negative emotions decreased it (Liu et al., 2021). Previous research has also shown that perceived teacher support can positively predict relaxation, and relaxation can positively predict self-efficacy among senior high school students (Liu et al., 2021). Given the possibility of gender differences in these impacts, the present study took gender as a covariate to extend this conclusion to junior high school students. The purpose of this study was to investigate whether academic emotions could play a mediating role between perceived teacher support and self-efficacy in the group of junior high school students.

#### The chain mediating role of achievement goals and academic emotions

SCT describes a dynamic interaction between perceived teacher support and self-efficacy (Bandura et al., 1997). Empirical research has shown that supportive messages and encouragement from teachers helped increase students’ effort and confidence in their learning (Liu et al., 2017). Students with high mastery goals are more likely to persist in their studies and invest more energy in them (Benita et al., 2014). However, students with mastery goals aim to improve the intrinsic value of their abilities rather than to demonstrate their abilities, so they tend to experience more positive emotions (e.g., pleasure, enjoyment) and fewer negative emotions (e.g., anxiety; Liu et al., 2020b). According to CVT, students will complete their tasks with confidence and generate positive emotions within themselves when they have a strong sense of control and value judgment over their given tasks (Pekrun, 2006; Liu et al., 2017). Similarly, a sense of control over a learning task may influence students’ motivations and interests, promoting the use of effective learning strategies and facilitating the pursuit of high academic goals (Liu et al., 2020b). Specifically, mastery oriented goals can positively predict students’ positive emotions (i.e., hope and pride) and negatively predict students’ negative emotions (i.e., boredom and anger; Pekrun, 2006). Academic emotions may trigger self-regulation and thus affect goal setting in CVT (Pekrun, 2006).

CVT suggests that achievement goals and academic emotions have a clear directional relationship (Pekrun, 2006). Moreover, the literature referenced in sections Achievement goals as the mediators and Academic emotions as the mediators also show that achievement goals and academic emotions can both mediate the relationship between perceived teacher support and self-efficacy. Still, there is a lack of current research on this specific relationship. Therefore, the authors supposed that achievement goals and academic emotions can play a chain mediating role between perceived teacher support and self-efficacy.

### The present study

The present study aimed to investigate whether achievement goals and academic emotions play a chain mediating role between perceived teacher support and academic self-efficacy, and therefore the following hypotheses were formulated:

*Hypothesis 1*: Perceived teacher support has a positive effect on junior high school students’ academic self-efficacy.

*Hypothesis 2*: Achievement goals play an intermediary role between perceived teacher support and academic self-efficacy.

*Hypothesis 3*: Academic emotions play an intermediary role between perceived teacher support and academic self-efficacy.

*Hypothesis 4*: Achievement goals and academic emotions play a chain intermediary role between perceived teacher support and academic self-efficacy.

## The Chinese education context

Recent shifts in national policies in China’s education sector have emphasized a focus on quality education and development. It is hoped that this may ease the burden of extra-curricular training on primary and secondary school students, reinstating the school campus as the center of learning once again. Many primary and secondary schools have already launched after-school extended-hours services following the policy change announcement, aiming to provide high-quality teaching services for students and to further promote the education reforms while reducing educational inequities across the education system (Wu, 2021). However, the gaps in education between the eastern and western regions of China, as well as between urban and rural areas, cannot be ignored. Particularly the gap between urban and rural education standards is a result of unequal investment in education, children’s educational attainment, school quality, and gaps in education (Zhang, 2017). The difference between the quality of available teachers at the compulsory education levels between urban and rural areas in particular has become a limiting problem in implementing or improving quality education in rural areas (Bao, 2005). Similar problems and gaps also exist between the eastern and western regions of the country due to differences in regional economic development.

As schools are the main setting for childhood education, there are high demands on teachers. Whether teachers can provide adequate teacher support for students in such an environment remains to be seen. When students lack adequate support, there is an increased risk of deviant behavior such as smoking, aggression, and drinking alcohol (Sakiz et al., 2012). Compared to their parents, teachers spend more time with their students, and as such, their words may have greater impact on students compared with their parents. Therefore, it is essential to explore the role of teacher support in student development.

## Materials and methods

### Participants and procedure

Participants in this study were secondary school students from seven junior high schools across eastern and western regions of China (*n* = 1,074, 501 females, *M_age_* = 12.66, *SD_age_* = 0.69, age range: 10–16 years, grades: 7–8). Data were collected through online surveys between 13 September and 19 October 2021. The questionnaires were uploaded to WJX,^1^ an online survey tool. In their information technology class, participants were informed about the study’s purpose, and a researcher directed them to complete the questionnaires online. Participants completed the questionnaires voluntarily and anonymously, taking them approximately 15 min.

### Measurements

#### Perceived Teacher Support Questionnaire

The Perceived Teacher Support Questionnaire was developed by Ouyang (2005) based on Babad’s (1990) research on teachers’ differential behaviors. Ten items were retained and the results showed an acceptable fit of the data (χ^2^/*df* = 4.11, RMSEA = 0.06, SRMR = 0.03, CFI = 0.97, and TLI = 0.96). Cronbach’s α was 0.90. This measure has demonstrated good reliability and validity among Chinese adolescents (Chen and Guo, 2016; Chen et al., 2018). This questionnaire measures students’ perceived attitudes regarding their teachers’ behaviors in supporting students in their academic studies at school, and includes three dimensions: academic support (three items, Cronbach’s α = 0.71, e.g., “My teacher thinks I’m always able to complete difficult homework or tasks”), emotional support (three items, Cronbach’s α = 0.73, e.g., “When I am answering questions, my teachers always stare me smilingly”), and competence support (four items, Cronbach’s α = 0.82, e.g., “My teachers always encourage me in learning and life”). Each item is scored on a six-point Likert scale. According to the description of the topic, the subjects rated it according its degree of similarity to their actual situation, from 1 (completely inconsistent) to 6 (completely consistent). The higher the score, the more support the student perceived from their teacher (Ouyang, 2005).

#### Achievement goals questionnaire

The Achievement Goals Questionnaire was adapted from Dong and Yu (2010), who based theirs on that of Elliot and McGregor (2001), to suit the research in the Chinese context. This questionnaire includes mastery-approach (three items, Cronbach’s α = 0.88, “It is important for me to do better than other students”), mastery-avoidance (three items, Cronbach’s α = 0.88, “Sometimes I’m afraid that I may not understand the content of this class as thoroughly as I’d like”), performance-approach (three items, Cronbach’s α = 0.79, “I want to learn as much as possible from this class”), and performance-avoidance (three items, Cronbach’s α = 0.82, “My fear of performing poorly in this class is often what motivates me).” The measure has demonstrated good reliability and validity among Chinese adolescents (Zhu et al., 2015). There are 12 items in total (Cronbach’s α = 0.85), which are scored using a seven-point Likert scale ranging from 1 (not at all true of me) to 7 (very true of me). The higher the score in a certain dimension, the more obvious the characteristics of this dimension to the student completing the questionnaire. The results of the confirmatory factor analysis (CFA) indicated an acceptable fit to the data (χ^2^/*df* = 2.96, RMSEA = 0.06, SRMR = 0.03, CFI = 0.97, and TLI = 0.96).

#### Academic emotions questionnaire

The Academic Emotions Questionnaire was adapted from the Academic Emotions Questionnaire for Adolescents compiled by Dong and Yu (2007). There are 27 items in the Academic Emotions Questionnaire for Adolescents, with four dimensions including positive high arousal, positive low arousal, negative high arousal, and negative low arousal. This measure has demonstrated good reliability and validity among Chinese adolescents (Dong and Yu, 2010; Gao, 2016). The adapted scale used in the current study had 21 items across the four dimensions of happy (four items, Cronbach’s α = 0.84, e.g., “I’m happy to get all items right”), relax (five items, Cronbach’s α = 0.88, e.g., “I can complete my study tasks with ease”), anxiety (four items, Cronbach’s α = 0.83, e.g., “In my study, I often encounter setbacks”), and tired (eight items, Cronbach’s α = 0.94, e.g., “I do not think it’s useful to study”), and the overall reliability was 0.65. The questionnaire is scored using a five-point Likert scale, with subjects scoring the items according to how well the items match their actual situation from 1 (very unlikely) to 5 (very likely). The higher the score, the more the student’s emotions match the measured dimension of emotions (Gai, 2019). CFA results indicated an acceptable fit to the data (χ^2^/*df* = 3.55, RMSEA = 0.05, SRMR = 0.06, CFI = 0.96, and TLI = 0.95).

#### Academic self-efficacy questionnaire

The Academic Self-Efficacy Questionnaire is a revised version of the Academic Self-Efficacy Questionnaire developed by Liang (2000), based on Pintrich and de Groot (1990). This measure has demonstrated good reliability and validity in Chinese adolescents (Chen et al., 2021; Wan et al., 2021). The present study selected 14 items including two dimensions of learning capability self-efficacy (nine items, Cronbach’s α = 0.93, e.g., “I believe I can get good grades in my studies”) and learning behavior self-efficacy (five items, Cronbach’s α = 0.85, e.g., “When studying, I always like to test whether I have mastered what I have learned by asking and answering myself”), with a total reliability of 0.94. The questionnaire is scored using a five-point Likert scale ranging from 1 (not at all) to 5 (exactly). The higher the score in a certain dimension, the more obvious the characteristic of this dimension (Li, 2020). CFA results indicated an acceptable fit to the data (χ^2^/*df* = 3.17, RMSEA = 0.05, SRMR = 0.03, CFI = 0.97, and TLI = 0.97).

#### Data analysis

First, invalid questionnaires (e.g., too short response time, serious age discrepancies in the completed questionnaire) were removed from the collected questionnaires before analyzing the data. After processing, the data was imported into SPSS 23.0. Then, the mean scores of each scale and its sub-dimensions were obtained separately, and the correlations between all variables and descriptive statistics were derived, as shown in Table 1. Next, CFA was conducted using Mplus 8.3 to analyze the structural validity of each dimension of the questionnaire. The significance of the mediating role was tested by bootstrapping 2,000 times (DiCiccio and Efron, 1996). In the current study, statistical significance was set at *p* < 0.05. There were three models used in this study, namely a model of achievement goals mediating perceived teacher support and self-efficacy, a model of academic emotions mediating perceived teacher support and self-efficacy, and a model in which achievement goals and academic emotions play a chain intermediary role between perceived teacher support and self-efficacy. To assess the fit of the three structural equation models (SEMs), the following indicators were used: the comparative fit index (CFI ≥ 0.90 was acceptable), Tucker-Lewis index (TLI ≥ 0.90 was acceptable), root mean square error of approximation (RMSEA < 0.06 was acceptable), and the standardized root mean residual (SRMR < 0.08 was acceptable); the indices that meet these requirements indicate a good model fit (Hu and Bentler, 1999). One point to note is that the value of χ^2^/*df* is large due to the relatively large sample size used in this study.

**Table 1:** Means, standardized deviation, and correlations for all variables.

Variable	1	2	3	4	5	6	7	8	9	10	11	12	13	14	15	16
1. Gender	—															
2. Academic support	0.02	—														
3. Emotional support	0.11^**^	0.72^**^	—													
4. Competence support	0.06^*^	0.68^**^	0.78^**^	—												
5. Performance-approach goal	0.00	0.22^*^	0.25^**^	0.27^**^	—											
6. Performance-avoidance goal	−0.03	0.01	0.01	0.03	0.26^**^	—										
7. Mastery-approach goal	0.08^**^	0.31^**^	0.40^**^	0.38^**^	0.56^**^	0.19^**^	—									
8. Mastery-avoidance goal	0.07^*^	−0.03	−0.01	−0.01	0.33^**^	0.42^**^	0.22^**^	—								
9. Happy	0.05	0.46^**^	0.52^**^	0.55^**^	0.34^**^	0.04	0.50^**^	0.04	—							
10. Relax	−0.09^**^	0.51^**^	0.54^**^	0.54^**^	0.28^**^	−0.07^*^	0.37^**^	−0.12^**^	0.60^**^	—						
11. Anxiety	0.15^**^	−0.07^*^	−0.06	−0.08^*^	0.09^**^	0.23^**^	0.06^*^	0.36^**^	0.00	−0.23^**^	—					
12. Tired	−0.01	−0.37^**^	−0.42^**^	−0.45^**^	−0.16^**^	0.15^**^	−0.38^**^	0.21^**^	−0.55^**^	−0.56^**^	0.30^**^	—				
13. Positive emotions	−0.03	0.55^**^	0.59^**^	0.61^**^	0.34^**^	−0.02	0.48^**^	−0.05	0.87^**^	0.91^**^	−0.14^**^	0.62^**^	—			
14. Negative emotions	0.10^**^	−0.26^**^	−0.28^**^	−0.31^**^	−0.04	0.24^**^	−0.18^**^	0.36^**^	−0.32^**^	−0.48^**^	0.83^**^	0.78^**^	−45^**^	—		
15. Learning ability	−0.11^**^	0.51^**^	0.53^**^	0.55^**^	0.36^**^	−0.03	0.47^**^	−0.09^**^	0.58^**^	0.71^**^	−0.24^**^	−0.52^**^	0.73^**^	−0.46^**^	—	
16. Learning behavior	0.02	0.46^**^	0.50^**^	0.49^**^	0.36^**^	0.02	0.50^**^	−0.01	0.51^**^	0.55^**^	−0.12	−0.46^**^	0.60^**^	−0.34^**^	0.74^**^	—
*M*	1.53	4.19	4.29	4.63	4.93	4.41	5.59	4.41	4.18	3.50	3.32	1.97	3.84	2.64	3.51	3.58
*SD*	0.50	1.05	1.05	1.03	1.25	1.52	1.21	1.48	0.70	0.84	0.97	0.85	0.69	0.73	0.73	0.73

M, mean; SD, standard deviation. All data converted to a standardized form (Z-score).

* *p* < 0.05;

** *p* < 0.01.

## Results

### Correlation analysis

Table 1 presents the correlation coefficients for all variables. The results reveal significant positive correlations among sub-dimensions of perceived teacher support, approach-oriented goals, positive emotions, and sub-dimensions of self-efficacy (0.22 < *r*s < 0.73, *p*s < 0.01). Negative emotions show significant negative correlations with mastery-approach goals and sub-dimensions of self-efficacy (−0.46 < *r*s < −0.18, *p*s < 0.01) and significant positive correlation with avoidance-oriented goals (0.24 < *r*s < 0.36, *p*s < 0.01). The mastery-avoidance goal also shows significant negative correlation with learning ability (*r*s < −0.09, *p*s < 0.01).

The specific model path coefficients and confidence intervals can be seen in Table 2, and the reliability and convergence validity of the measurement model in Table 3.

**Table 2:** Path coefficients and confidence intervals of all three models.

Model	Effect	Significance test of parameters				95% confidence level	
		β	S.E.	Est./S.E.	*p*	LLCI	ULCI
Model 1	**Direct effect**						
	PTS-SE	0.52^***^	0.03	15.56	0.00	0.46	0.59
	**Indirect effect**						
	PTS-PPG-SE	0.07^***^	0.02	4.13	0.00	—	—
	PTS-PVG-SE	—	0.01	0.56	0.58	—	—
	PTS-MPG-SE	0.12^***^	0.02	5.10	0.00	—	—
	PTS-MVG-SE	—	0.00	−0.14	0.89	—	—
Model 2	**Direct effect**						
	PTS-SE	—	0.13		0.41	−0.40	0.10
	**Indirect effect**						
	PTS-PE-SE	0.77^***^	0.13		0.00	—	—
	PTS-PE-SE	—	0.07		0.39	—	—
Model 3	**Direct effect**						
	PTS-SE	—	0.24		0.42	−0.77	−0.01
	**Indirect effect**						
	PTS-PPG-PE-SE	—	0.02		0.06	—	—
	PTS-PPG-NE-SE	—	0.01		0.86	—	—
	PTS-PVG-PE-SE	—	0.01		0.42	—	—
	PTS-PVG-NE-SE	—	0.01		0.83	—	—
	PTS-MPG-PE-SE	0.11^**^	0.04		0.01	—	—
	PTS-MPG-NE-SE	—	0.08		0.62	—	—
	PTS-MVG-PE-SE	—	0.00		0.94	—	—
	PTS-MVG-NE-SE	—	0.00		0.97	—	—
—	**Direct effect**						
	PTS-PPG	0.31^***^	0.04		0.00	0.34	0.57
	PTS-PVG	—	0.04		0.18	−0.20	0.04
	PTS-MPG	0.45^***^	0.03		0.00	0.55	0.81
	PTS-MVG	—	0.04		0.83	−0.13	0.11
	PTS-PE	0.80^***^	0.03		0.00	0.74	0.85
	PTS-NE	−0.53^***^	0.07		0.00	−0.65	−0.39

PTS, Perceived teacher support; PPG, Performance-approach goal; PVG, Performance-avoidance goal; MPG, Mastery-approach goal; MVG, Mastery-avoidance goal; PE, Positive emotion; NE, Negative emotion; SE, Self-efficacy; LLCI, bootstrap sampling 95% interval lower limit; ULCI, bootstrap sampling 95% interval upper limit.

** *p* < 0.01;

*** *p* < 0.001.

**Table 3:** The reliability and convergence validity of the measurement model.

Measurement model			Significance test of parameters				Item reliability	Composite reliability	Convergence validity
			Estimate	S.E.	Est./S.E.	*p*	R^2^	CR	AVR
PTS	PTAS	A1	0.70	0.02	32.43	***	0.49	0.91	0.51
		A2	0.78	0.02	45.92	***	0.60		
		A3	0.57	0.03	22.55	***	0.33		
	PTES	A4	0.68	0.02	37.30	***	0.46		
		A5	0.61	0.02	28.03	***	0.38		
		A6	0.78	0.02	52.70	***	0.61		
	PTCS	A7	0.86	0.01	76.55	***	0.74		
		A8	0.66	0.02	29.37	***	0.43		
		A9	0.73	0.02	38.92	***	0.53		
		A10	0.70	0.02	37.87	***	0.50		
AG	PPG	A11	0.73	0.02	33.29	***	0.53	0.96	0.65
		A12	0.71	0.02	29.56	***	0.50		
		A13	0.81	0.02	40.32	***	0.65		
	PVG	A14	0.77	0.02	37.93	***	0.59		
		A15	0.91	0.01	79.82	***	0.83		
		A16	0.85	0.02	51.21	***	0.71		
	MPG	A17	0.84	0.02	52.14	***	0.70		
		A18	0.91	0.01	84.98	***	0.83		
		A19	0.80	0.02	47.45	***	0.64		
	MVG	A20	0.73	0.02	30.43	***	0.53		
		A21	0.87	0.02	45.32	***	0.76		
		A22	0.74	0.02	33.08	***	0.55		
AE	Happy	A23	0.68	0.02	29.35	***	0.46	0.97	0.62
		A24	0.74	0.02	41.72	***	0.55		
		A25	0.82	0.02	49.62	***	0.68		
		A26	0.80	0.02	44.71	***	0.65		
	Relax	A27	0.81	0.01	56.69	***	0.65		
		A28	0.75	0.02	47.80	***	0.56		
		A29	0.76	0.02	47.89	***	0.58		
		A30	0.82	0.01	67.91	***	0.67		
		A31	0.74	0.02	38.46	***	0.55		
	Anxiety	A32	0.66	0.02	27.03	***	0.44		
		A33	0.71	0.02	36.14	***	0.50		
		A34	0.83	0.02	46.83	***	0.68		
		A35	0.78	0.02	44.76	***	0.60		
	Tired	A36	0.79	0.02	44.35	***	0.62		
		A37	0.82	0.02	54.54	***	0.67		
		A38	0.90	0.01	76.10	***	0.80		
		A39	0.77	0.02	51.20	***	0.59		
		A40	0.86	0.01	77.73	***	0.73		
		A41	0.74	0.02	50.19	***	0.54		
		A42	0.89	0.01	89.61	***	0.79		
		A43	0.83	0.01	62.75	***	0.70		
SE	LA	A44	0.73	0.02	45.54	***	0.54	0.95	0.58
		A45	0.77	0.02	51.35	***	0.59		
		A46	0.76	0.01	55.75	***	0.58		
		A47	0.85	0.01	91.87	***	0.72		
		A48	0.80	0.01	59.02	***	0.63		
		A49	0.74	0.02	50.61	***	0.55		
		A50	0.77	0.01	57.41	***	0.59		
		A51	0.82	0.01	65.46	***	0.68		
		A52	0.74	0.02	49.22	***	0.54		
	LB	A53	0.69	0.02	35.67	***	0.48		
		A54	0.82	0.01	63.02	***	0.67		
		A55	0.73	0.02	40.14	***	0.53		
		A56	0.75	0.02	46.08	***	0.57		
		A57	0.70	0.02	35.73	***	0.49		

PTS, perceived teacher support; PTAS, perceived teacher academic support; PTES, perceived teacher emotional support; PTCS, perceived teacher competence support; AG, achievement goal; PPG, performance-approach goal; PVG, performance-avoidance goal; MPG, Mastery-approach goal; MVG, mastery-avoidance goal; AE, academic emotion; SE, self-efficacy; LA, learning ability; LB, learning behavior.

*** *p* < 0.001.

### Directed effect

**Figure 1:**
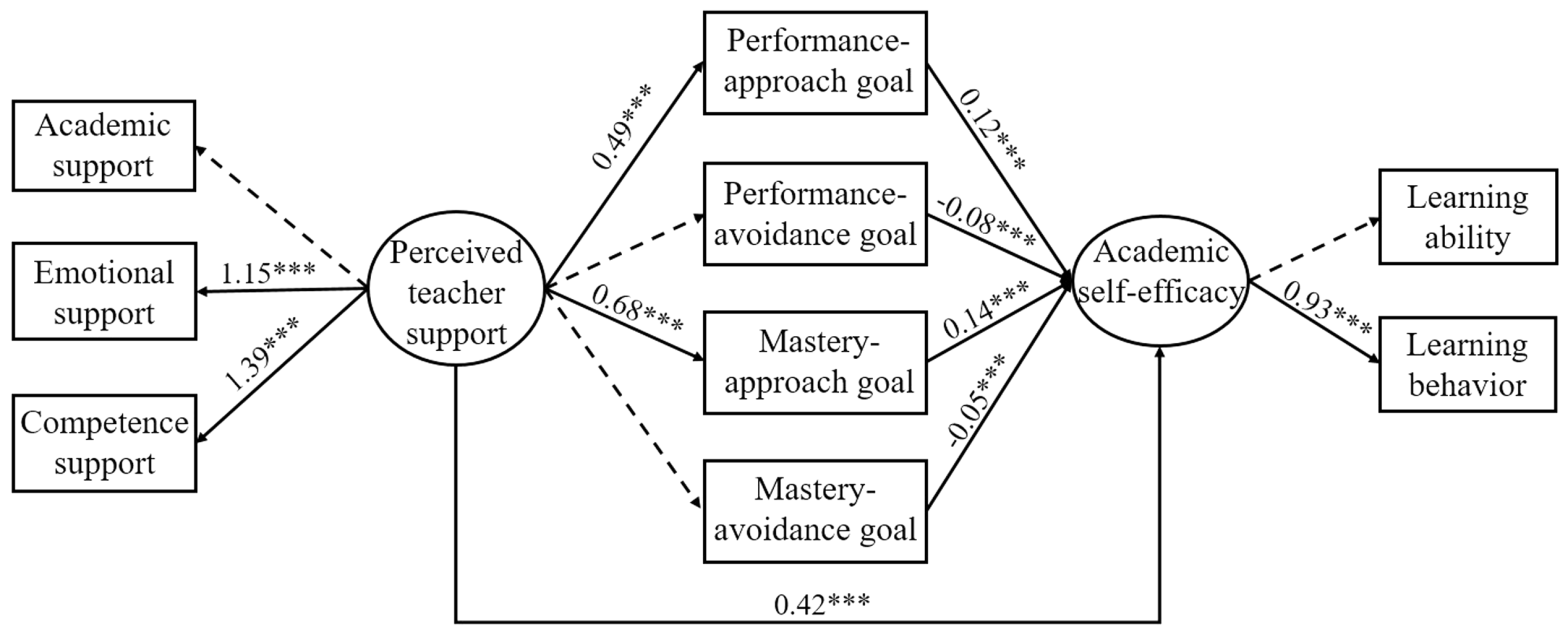
SEM of achievement goals as the mediators. The coefficients shown are standardized path coefficients. A solid arrow represents a significant path, and a dotted arrow represents an insignificant path (^***^p < 0.001).

**Figure 2:**
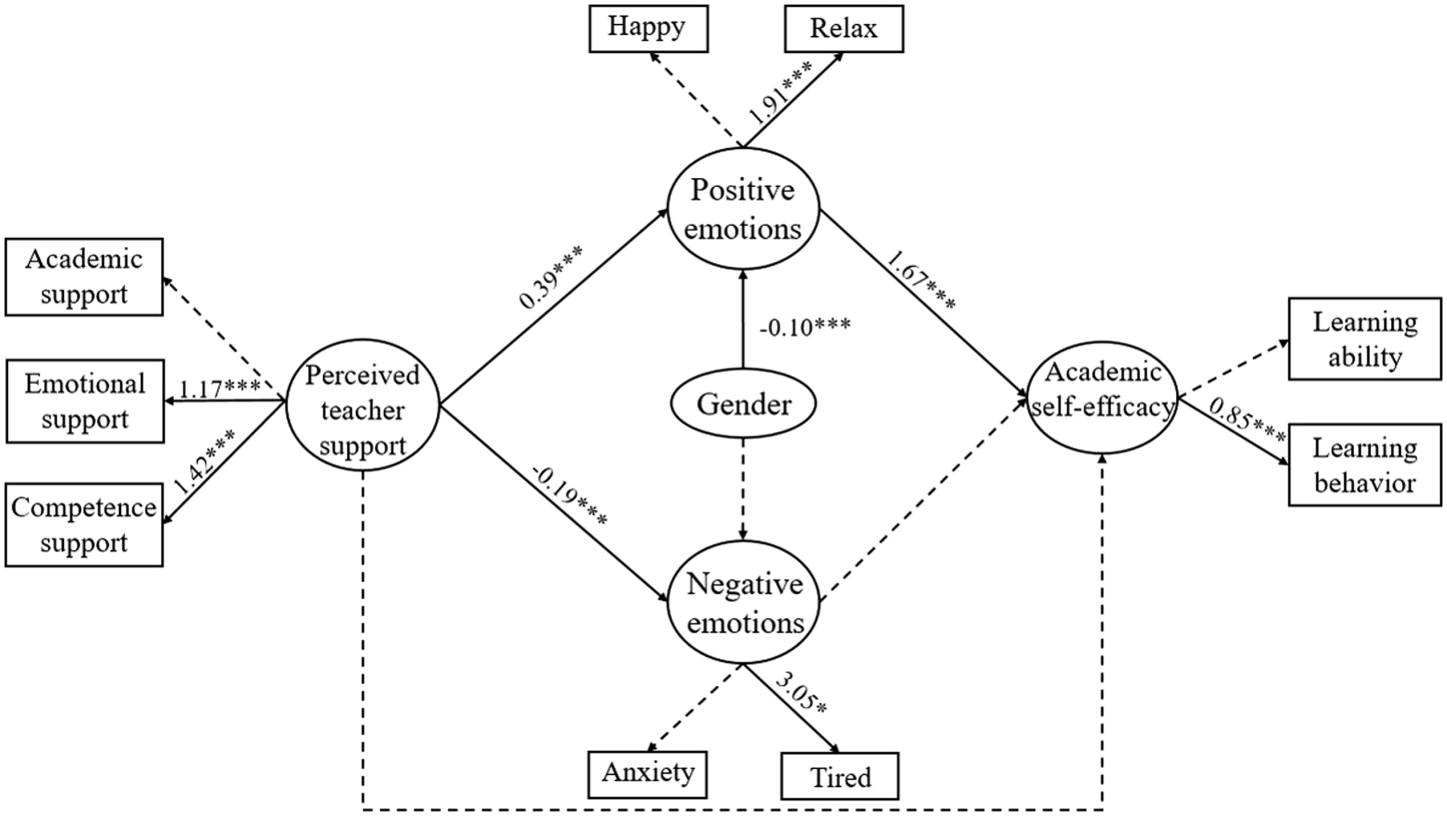
SEM of academic emotions as mediators. The coefficients shown are standardized path coefficients. A solid arrow represents a significant path, and a dotted arrow represents an insignificant path (^*^p < 0.05, ^***^p < 0.001).

**Figure 3:**
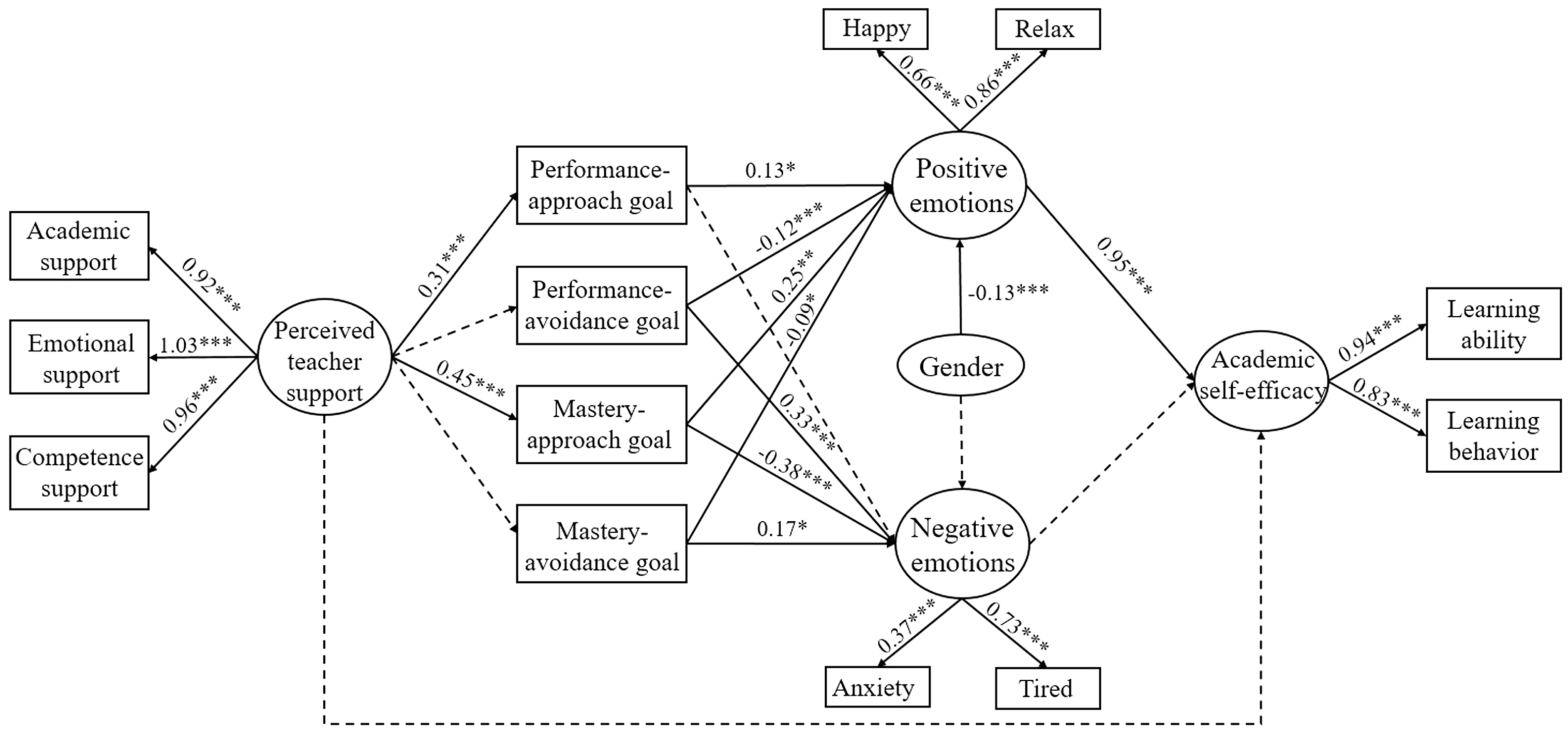
Multiple mediation model. The coefficients shown are standardized path coefficients. A solid arrow represents a significant path, and a dotted arrow represents an insignificant path (^*^p < 0.05, ^**^p < 0.01, ^***^p < 0.001).

A peculiar phenomenon is that the relationship between perceived teacher support and self-efficacy is inconsistent across the three models used in this study: Figure 1 shows how teacher support positively predicted self-efficacy (*β* = 0.52, *p* < 0.001, 95%CI [0.46, 0.59]) and verified Hypothesis 1; while the paths between teacher support and self-efficacy were not significant in Figures 2, 3, which did not support Hypothesis 1. When achievement goal is a mediator, the direct influence between perceived teacher support and academic self-efficacy is significant. When academic emotions are used as mediating variables, the results are opposite. The direct role between perceived teacher support and self-efficacy is also not significant n the chain mediation model, which may be caused by the addition of academic emotions.

### SEM of achievement goals as the mediators

To ensure conciseness in the model, all insignificant path coefficients and confidence intervals were deleted from the initial model. As shown in Figure 1, the path between learning support and perceived teacher support is not significant, which is the same as the relationship between learning ability and self-efficacy. However, this did not affect the construction of the present model and the data analysis showed that the model fit well (CFI = 0.92, TLI = 0.91, RMSEA = 0.06, SRMR = 0.08, χ^2^/*df* = 4.21). As seen in Table 2, perceived teacher support also positively predicted changes in self-efficacy by performance-approach goal or mastery-approach goal (*β* = 0.53, *p* < 0.001; *β* = 0.12, *p* < 0.001). In other words, approach-oriented goals can play a mediating role between perceived teacher support and self-efficacy, which partially supported Hypothesis 2.

### SEM of academic emotions as mediators

Some paths between the explicit and latent variables in this model were not significant, which caused the range of measurable variables to be smaller, but the overall model fit was good (CFI = 0.93, TLI = 0.93, RMSEA = 0.05, SRMR = 0.06, χ^2^/*df* = 3.19). Because gender affected academic emotions, it was modeled as a covariate. The results showed that gender had negative predictive role on positive emotions (*β* = −0.14, *p* < 0.001) but no significant influence on negative emotions. The aforementioned results suggest that there was no direct impact between perceived teacher support and self-efficacy, and perceived teacher support significantly positively predicted changes in self-efficacy through positive emotions (*β* = 0.77, *p* < 0.001). This finding partly supported Hypothesis 3.

### Chain mediation model

After verifying the mediating role of achievement goals and academic emotions between perceived teacher support and self-efficacy –4.1 and 4.2, respectively—the chain mediation model was constructed which showed good fit (CFI = 0.93, TLI = 0.92, RMSEA = 0.04, SRMR = 0.07, χ^2^/*df* = 2.83). As seen in Figure 3 and Table 2, two of the eight possible paths in the chain mediation model were statistically significant. Mastery-approach goals and positive emotions were shown play a chain mediating role between perceived teacher support and self-efficacy (*β* = 0.11, *p* < 0.001). The path of perceived teacher support positively predicting self-efficacy through performance-approach goals and positive emotions is borderline significant (*p* = 0.06). The two paths together supported Hypothesis 4.

## Discussion

The present study aimed to examine the mediating roles of achievement goals and academic emotions in the association between perceived teacher support and self-efficacy. First, the results showed that perceived teacher support directly predicted approach-oriented goals and positive emotions positively and negatively predicted negative emotions. However, the direct role between perceived teacher support and self-efficacy differed across contexts Second, perceived teacher support positively predicted self-efficacy by positively predicting approach-oriented goals or positive academic emotions. Further research indicated that perceived teacher support positively predicted approach-oriented goals and further positively predicted self-efficacy by positively predicting changes in positive affect.

### Understanding the two measurement models

#### SEM of achievement goals as mediators

First, the results showed that the more that students perceived support from their teacher, the more likely the students were to focus on approach-oriented goals. Support from teachers can enhance students’ self-confidence in completing tasks and motivate them to work hard to accomplish goals that can be obtained through efforts (Metheny et al., 2008). Second, perceived teacher support can positively directly predict self-efficacy and indirectly predict self-efficacy by positively predicting approach-oriented goals. According to the principles of SDT theory, support from teachers can motivate students to learn (Ciani et al., 2011) as the individual wants to respond to the teacher’s support subconsciously (i.e., show that they can do better). When motivated by approach-oriented goals, individuals are more likely to perform even better. Furthermore, when students achieve outcomes that are not too far from their expectations, they develop a good sense of self-efficacy in that they are aware of what they are capable of doing, which provides feedback in response to the teacher’s support and thus forms a virtuous circle. Meanwhile, avoidance-oriented goals do not play a mediating role between perceived teacher support and self-efficacy. Students who endorse avoidance-oriented goals may have a more conservative personality and their goal-setting is such that they are attempting to avoid failure (Van Yperen et al., 2009). Therefore, even if they do receive support from their teachers, it can be difficult for them to change the routines and expectations which have been developed through their own personality traits. One study that used an achievement goals trisection showed that individuals were more intrinsically motivated when they adopted mastery goals, while adopting performance-avoidance goals led to lower intrinsic motivation (Benita et al., 2014). Future research should be done considering motivation and personality characteristics to further refine our understandings of these mechanisms.

#### SEM of academic emotions as mediators

First, the results showed that support from teachers can stimulate positive emotions in students. However, when students lacked teacher support, students were prone to develop negative emotions such as anxiety and tiredness. These two findings are consistent with those of previous research (Lei et al., 2017; Zhen et al., 2017). Furthermore, studies have shown that females tended to receive more teacher support than males in social settings (Baumeister and Sommer, 1997). Therefore, we included gender as a covariate in our model. The results showed that although there were no differences in perceptions of negative emotions between male and female students, males were less sensitive than females at perceiving positive emotions. Overall, when students perceived more teacher support, they were more likely to produce positive emotions and feel more self-efficacy. According to the broaden-and-build theory, when individuals develop positive attitudes, their self-efficacy is also enhanced, which has also been supported by previous research findings (Liu et al., 2021). However, negative emotions were not shown to play a mediating role between perceived teacher support and self-efficacy in the current study. Specifically, perceived teacher support could be seen to predict the change of negative emotions, but there was no correlation between negative emotions and self-efficacy. However, there may be a nonlinear relation between positive emotions and self-efficacy. According to CVT, positive emotions may elicit intrinsic motivation, while extrinsic motivation may conversely be induced to avoid failure (Pekrun, 2006).

### Understanding the chain mediation model

The Pygmalion effect states that when students receive support from their teachers, the students tend to set higher goals and work harder to accomplish them (Szumski and Karwowski, 2019). Indeed, studies have shown that students’ internal motivation was awakened when receiving support from teachers (Linnenbrink and Pintrich, 2019). Students become more confident that they can reach their set goals and produce positive academic emotions, which in turn affect change in their self-efficacy. Inconsistent with previous studies, as well as the findings of the current study, Benita et al. (2014) believed that mastery goals led to more positive emotional experiences in the context of autonomy support. This alternative conclusion may be due to the fact that Benita et al. (2014) used ternary goal classification for achievement goals without further refinement of mastery goals, whereas the present study used a 2 × 2 goal classification. Therefore, we consider the findings of Benita et al. as being complementary to the findings regarding the relationship between mastery goals and academic emotions. Furthermore, the current study found no correlation between perceived teacher support and negative emotions, which is consistent with the conclusions of many previous studies (Pekrun et al., 2009). The lack of correlation may be because students with performance-approach goals focus on surpassing others and maintaining high social status, meaning that goal orientation does not correlate with negative emotions (Sideridis, 2005).

### Implications

#### Theoretical implications

Many empirical studies have already explored the mechanism of action between perceived teacher support and self-efficacy, but none thus far have detailed a complete, systematic model of it. Therefore, the current study aimed to contribute by developing a systematic model. Furthermore, as the junior high school years are an important stage in the developmental process of students, this study chose to verify the relationship between perceived teacher support and self-efficacy in the context of junior high school students. The findings confirmed that both achievement goals and academic emotions mediate the relationship between perceived teacher support and self-efficacy. And it was further extended to find that achievement goals and academic emotions can play a chain intermediary role between perceived teacher support and self-efficacy. These findings enrich the understanding of the mechanisms at play in the promotion of student learning in the Chinese educational context, and can potentially be generalized to include other Asian cultures as well.

#### Practical implications

This study was conducted in a period of normalization during the COVID-19 pandemic and amidst changes to education policies in China, when this unique intersection poses greater challenges for school education. During the pandemic, students may have been feeling less safe and requiring more external support. However, it has been difficult for teachers to monitor their students’ situations in distance learning, and the process of students seeking help from their teachers has become more convoluted. Moreover, changes in education policies removed an additional learning scaffold from some students who had relied on extracurricular classes, which may have increased their academic anxiety. Despite the availability of after-school extended-hours services, it is still uncertain how students will adapt to the new system, which will require further exploration. The current study focused on this phenomenon by delving into the mechanism of action between perceived teacher support and self-efficacy. The findings can help teachers better understand this problem and be aware of their students’ needs and adjust their teaching behaviors accordingly. Teachers can understand students’ learning status through various channels (regular surveys, strengthening communication between home and school, etc.) and provide timely assistance to students. In addition, teachers can have students plan for short- and long-term goals and help them adjust their goals to approach-oriented goals. And in real time, students will be asked to record their psychological status and changes in self-efficacy in the form of self-reports. On the one hand, the implementation of the above measures must be systematized and consistent. On the other hand, effective supervision by teachers is needed. To achieve fine management, certain timely or effective management models can be introduced as appropriate. How to leverage student motivation to drive the entire model is something that educational practitioners need to explore in the course of their practice. We hope that this study will alert educational leaders and policymakers to this phenomenon so that they can develop systematic measures to better serve students.

## Limitations and future directions

The present study provides empirical evidence for the influences of teacher support on student learning and development, and can facilitate the further construction of a systematic model related to the variables in this study, but there are still several limitations. First, this study is a cross-sectional study, which can only verify the existence of mediating influence is among variables and cannot prove the causal relationship between variables. In addition, many recent studies based on CVT have demonstrated that self-efficacy can predict academic emotions and, in turn, that academic emotions can predict changes in self-efficacy (Pajares, 1997; Pekrun, 2006; Liu et al., 2017, 2021). Therefore, a longitudinal study could be considered to explore the causal relationship between variables in terms of their bidirectional impacts. Second, this study focused only on the negative role of negative emotions. However, negative emotions can also have positive role, which this study did not analyze in detail. Thus, future research should further refine the dual role of negative emotions. Third, this study used self-report questionnaires to determine the academic emotions of junior high school students, which can be impacted by the self-expectancy effect; as such, the objectivity of the results obtained must be enhanced. Future research should incorporate more objective observation methods such as functional magnetic resonance imaging (fMRI) and electroencephalogram (EEG) to increase the objectivity of the data. Finally, the influences of teacher support on students can change depending on the student’s age and grade (Tao et al., 2022). Therefore, it is also necessary to consider students’ grade as an object of investigation in future research.

Despite these limitations, the authors hope that the findings of this study offer insight into how students can set more attainable goals, and how they can better achieve them through a “bounce” of support from their teachers. Teachers can consciously mobilize their students’ positive emotions to help them maintain a positive learning state. When students have a good sense of self-efficacy, they will be better able to accurately “predict” their abilities and thus improve their goal-setting skills, allowing them to build their confidence by reaching these goals, even when their aim is an A+.

## Data availability statement

The raw data supporting the conclusions of this article will be made available by the authors, without undue reservation.

## Author contributions

CW contributed to the study’s conception and design and performed the material preparation and data collection. BJ and HL performed data analysis. The first draft of the manuscript was written by XR and BJ. XR, BJ, HL, and CW commented on previous versions of the manuscript. All authors contributed to the article and approved the submitted version.

## Funding

This work was supported by the school level project of Sichuan Normal University (2022), research on the image design of pedagogical agent and its cognitive mechanism on learning (22XW070).

## Conflict of interest

The authors declare that the research was conducted in the absence of any commercial or financial relationships that could be construed as a potential conflict of interest.

## Publisher’s note

All claims expressed in this article are solely those of the authors and do not necessarily represent those of their affiliated organizations, or those of the publisher, the editors and the reviewers. Any product that may be evaluated in this article, or claim that may be made by its manufacturer, is not guaranteed or endorsed by the publisher.

## Footnotes

^1^ www.wjx.cn
